# Magneto-Tunable Surface Roughness and Hydrophobicity of Magnetoactive Elastomers Based on Polymer Networks with Different Architectures

**DOI:** 10.3390/polym17172411

**Published:** 2025-09-04

**Authors:** Sobit E. Kirgizov, Sergey A. Kostrov, Elena Yu. Kramarenko

**Affiliations:** 1 Engineering Physics Institute, Samarkand State University named after Sharof Rashidov, Samarkand 140104, Uzbekistan; qirgizovsobit94@gmail.com; 2 Faculty of Physics, Lomonosov Moscow State University, 119991 Moscow, Russia; 3 A.N. Nesmeyanov Institute of Organoelement Compounds of the Russian Academy of Sciences (INEOS), 119334 Moscow, Russia; sergeykostrov1996@gmail.com; 4 Enikolopov Institute of Synthetic Polymeric Materials, Russian Academy of Sciences, 117393 Moscow, Russia

**Keywords:** magnetoactive elastomer, surface roughness, hydrophobicity, water contact angle, polymer network, magnetorheological effect

## Abstract

In this study, we present experimental investigations of the surface structure and water contact angles of magnetoactive elastomers (MAEs), which are controlled by an external magnetic field. Specifically, we examine how the polymer matrix architecture affects the surface roughness and wettability of MAEs in various magnetic fields. We performed a comparative analysis on MAEs based on a linear polysiloxane network and on a matrix of the same chemical nature containing side-grafted chains. We synthesized a series of magnetoactive elastomers containing 75 wt.% carbonyl iron and varying amounts of a low-molecular-weight plasticizer. Although the magnetorheological effect is higher for traditional linear MAEs, we found that the magnetic response in surface properties is higher for novel MAEs with side-grafted chains. The largest increase in water contact angle was observed in the side-chain MAEs with the highest 60 wt.% plasticizer content: rising from 112° in a zero field to 168° in a 490 mT magnetic field. Water contact angles exhibit greater stability over time for side-chain MAEs, and this stability further increases in the presence of a magnetic field. Our results demonstrate that the architecture of the polymer matrix serves as an effective tool for designing smart, magnetically responsive surfaces.

## 1. Introduction

Magnetoactive elastomers (MAEs), also referred to as magnetorheological elastomers, represent a class of smart composite materials composed of magnetic microparticles dispersed within a polymeric matrix [1,2,3,4,5,6,7,8]. These materials combine the elastic properties of the soft matrix with the magnetic responsiveness of the embedded filler, enabling dynamic control over their mechanical and physical behavior under an external magnetic field. The application of a magnetic field induces particle magnetization, interparticle interactions, and reorganization of the magnetic particles into anisotropic structures [5,6]. The off-field mechanical properties of MAEs, particularly the elastic modulus of the polymer matrix, play a crucial role in determining the extent of particle mobility and subsequent field-induced transformations. In soft MAEs, where the matrix offers minimal resistance to particle motion, even weak magnetic fields can trigger substantial rearrangements of the magnetic particles [5,6,9,10]. These structural changes, in turn, influence various macroscopic properties depending on the particle distribution within the polymer matrix, including the material’s viscoelasticity, and magnetic and electric properties, as well as deformation behavior [10,11,12,13]. Due to these tunable characteristics, MAEs are considered promising candidates for applications in various fields, including medicine [14,15,16], soft robotics [17,18,19,20], adaptive structures, and sensor technologies [21,22].

In addition to changes in bulk properties, MAEs have been shown to exhibit reversible surface topography changes in response to magnetic stimulation. Recent studies have demonstrated that exposure to an external magnetic field can induce the formation of “mountain-like” or “needle-like” surface structures on MAE surfaces, resulting from the alignment of magnetic particles along the direction of the applied field, which is oriented perpendicular to the surface [23,24,25,26]. This phenomenon enables rapid and reversible modulation of surface roughness, adhesion, and friction, with reported sensitivity values of up to 1–10 µm/T in terms of root mean square surface height deviation per unit magnetic field [25,27,28,29,30,31]. Such responsiveness has sparked growing interest in the study of MAE surface properties and their use in tunable surface engineering [32,33,34,35,36,37,38,39,40].

Notably, the increase in surface roughness induced by magnetic fields has been linked to enhanced hydrophobicity, making MAEs attractive for applications that require dynamic control of wetting properties [23,24,25,28]. The underlying mechanism involves the formation of microscale protrusions on the surface, which alter the effective contact angle and mimic the well-known lotus effect. The geometry and extent of these surface features depend on several factors, including the concentration, size, and shape of the magnetic particles, as well as the mechanical properties of the polymer matrix [3,30,31,32,33], many of which have not yet been fully explored. Therefore, further research in this direction is essential for the development of magnetically responsive hydrophobic surfaces based on MAEs.

This paper presents the results of an analysis of the effect of polymer matrix architecture on the surface relief and water contact angles of MAEs. MAEs based on two different silicone matrices were synthesized and investigated. The first type of MAE was prepared using a commercially available compound forming a linear-chain polymer network, while the second type was based on a matrix containing side chains. Although both matrices exhibited similar initial elastic moduli, this similarity arose from different underlying factors: the first matrix contained a higher amount of low-molecular-mass plasticizer, whereas the lower modulus of the second matrix resulted from the presence of elastically inactive side chains that effectively dilute the network structure. It should be noted that a special class of elastomers with a high side-chain grafting density—so-called bottlebrush elastomers—can exhibit an extremely low elastic modulus of approximately 100 Pa, even in the absence of solvent [41,42,43]. It has previously been shown that side-chain MAEs display a significant magnetorheological effect even when free of, or containing minimal amounts of, low-molecular-weight components—which are prone to leaching both spontaneously and under mechanical deformation—and thus demonstrate superior performance [44,45,46,47].

A comparative analysis was conducted to evaluate the magnetic response in terms of surface roughness and wettability for MAEs based on these two polymer networks. All samples had a fixed concentration of magnetic filler. The content of the additionally added low-molecular-mass plasticizer was varied from 25 to 60 wt.%. We demonstrated that the presence of side chains in the polymer network significantly enhances the surface response to an applied magnetic field. Furthermore, we established a correlation between changes in the water contact angle and the induced surface roughness. In addition, we investigated how the contact angle depends on the thickness of the MAE sample and the concentration of the magnetic filler. Finally, we examined the time dependence and hysteresis of the contact angle under magnetic field application and removal. The results demonstrate significant advantages of using the developed materials with a partially branched matrix architecture in enhancing the magnetic responsiveness of surface properties.

## 2. Materials and Methods

### 2.1. Materials

In this study, two types of silicone elastomer matrices are used for the preparation of MAEs. The first one (Matrix 1) is prepared from the following five components: 7% hexachloroplatinic acid solution in isopropyl alcohol (Speier’s catalyst, “Sigma Aldrich”, Darmstadt, Germany); α,ω-divinylpolydimethylsiloxane (DVK-5 brand, “Penta-91”, Moscow, Russia); polymethylhydrosiloxane (P-804 brand, “Penta-91”, Moscow, Russia); ω-vinylpolydimethylsiloxane; and α,ω-dihydridepolydimethylsiloxane synthesized according to the method described in detail in [44].

The second matrix (Matrix 2) is based on a commercial two-component compound “SilcoPlat” (“ChimSnab”, Moscow, Russia). The two main components of this compound are vinyl- and hydride-containing silicones with the molecular mass of M_n_ = 70,000, M_w_ = 100,000. The compound also contains some amount of silicone oil (M_n_ = 5000, M_w_ = 7800) and aerosil.

The characteristics of the components and the composition of both types of the matrices are presented in Table 1.

Low-molecular-weight silicone oil (PMS-50, “Penta 91”, Moscow, Russia) is used as a plasticizer in both types of elastomers to tune the elastic modulus of the resulting material.

Spherical carbonyl iron particles (CIP) with an average diameter of 4.5 μm, purchased from «Spektr-Khim» (China), are used as magnetic filler particles. CIP are characterized by a high permeability and high saturation magnetization that are important for the MAE performance [5], in particular, for an effective surface properties control by magnetic fields [23,24,25].

### 2.2. Synthesis of MAE Samples

Both types of polymer matrices contain vinyl and hydride functional groups, which form covalent bonds in the presence of a platinum-based catalyst according to the following reaction:

$$\sim Si-CH=CH_{2}+H-Si\sim \overset{\;Pt\;}{\to }\sim Si-CH_{2}-CH_{2}-Si\sim$$

The hydrosilylation reaction proceeds without generating by-products, which is one of the advantages of using silicone-based compositions for producing high-performance magnetoactive elastomers.

**Figure 1:**
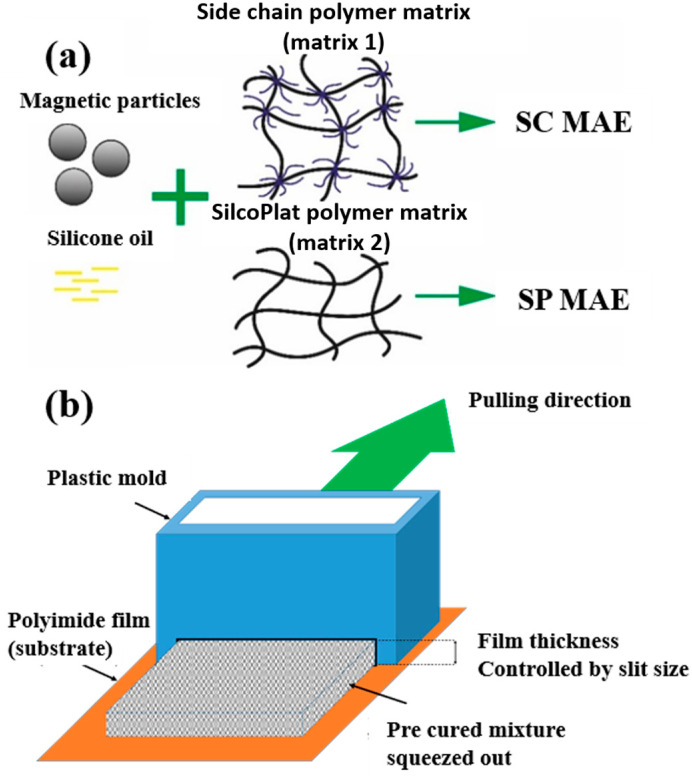
Components for synthesis of MAEs based on the side-chain polymer matrix (SC MAE) and on SilcoPlat matrix (SP MAE) as indicated in the figure (**a**). Schematic presentation of MAE film preparation (**b**).

The presence of a monofunctional component—ω-vinyl-polydimethylsiloxane with a single vinyl end group—in the composition of Matrix 1 results in the formation of side chains at the crosslinking points of the resulting network, as illustrated in Figure 1a. To emphasize the presence of side chains in this matrix, we refer to it as the side-chain matrix, or SC matrix. In contrast, the commercial SilcoPlat polymer matrix (abbreviated as SP matrix, after the trade name) forms a network composed of linear network strands, as schematically shown in Figure 1a.

The procedure for preparing samples for measurement is as follows. First, all polymer components are mixed together in a beaker. Then, silicone oil and magnetic particles are added to the mixture (Figure 1a) and the mixture is mechanically mixed. The SP-type compound already contains a platinum catalyst in one of its components, while for the preparation of SC polymer matrix, a solution of Spier’s catalyst is added at a ratio of 10 μL of solution per 1 g of polymer, which is then mechanically mixed again. To remove air bubbles formed during mixing, the beaker is placed in a vacuum chamber at a pressure of ~ 1 mm Hg for 2 min. At this stage, the mixture is ready for curing. To prepare thin films for contact angle measurements, the mixture is poured into a 3D-printed mold with no bottom surface and a narrow slit at the bottom of one wall. This mold is then slid smoothly in the direction opposite to the slit-wall orientation, which slowly squeezes the mixture out of the mold to form a film whose thickness is controlled by the size of the slit set to 0.2 and 0.4 mm (Figure 1b). The film in the mold is then placed in an oven for curing at 90 °C. The disk-shaped samples with a diameter of 20 mm and a height of about 1 mm for the magnetorheological measurements are prepared in a similar way by pouring the compound mixture into the mold of the prescribed size.

For each type of elastomer matrix, MAEs containing 75 wt.% CIP are prepared with four different mass concentrations of silicone oil (0%, 25%, 40%, and 60%). In addition, SC-matrix samples with 65 and 70 wt.% of CIP and 0, 25% of oil are synthesized. The CIP concentration is calculated relative to the total sample mass, while the oil concentration is calculated relative to the mass of the elastomer.

### 2.3. Methods

GPC was performed on a chromatographic system including a Stayer s.2 high-pressure pump (Akvilon, Moscow, Russia), a Smartline RI 2300 refractometer (Knauer, Berlin, Germany), and a Jetstream 2 plus thermostat (Knauer, Berlin, Germany). Analysis conditions: temperature, 40 ± 0.1 °C; eluent, toluene + 2% THF; flow rate, 1.0 mL/min; columns, 300 × 7.8 mm^2^; 5 μm Phenogel sorbent (Phenomenex, Torrance, CA, USA); pore size, 10^3^–10^5^ Å.

The shear modulus of all obtained MAEs was measured with the use of the rheometer Anton Paar Physica MCR 302 (“Anton Paar GmbH”, Graz, Austria) in the dynamic mode of harmonic torsion oscillations in the plate–plate measuring cell. The disk-like samples of the diameter 20 mm and the height of approximately 1 mm were placed between a stationary bottom plate and the upper plate connected to the rotor. The measurements were performed in the regime of linear viscoelasticity at the constant frequency of 10 rad/s and the strain amplitude of 0.001. The temperature was kept constant, equal to 20 °C.

Microscopy experiments were performed using a JEOL (Tokyo, Japan) JSM-6000PLUS benchtop scanning electron microscope (SEM).

Images of the MAE film surface in the absence and in the presence of magnetic field oriented perpendicular to the sample surface were obtained by Zeiss Axioskop 40 optical microscope.

The surface structure of MAE films was investigated using MicroXAM-100—3D Surface Profilometer and Optical Interferometer (“KLA Instruments”, Milpitas, CA, USA). It allowed us to get digital images of a surface and its roughness without any mechanical contact with the surface. The image of the surface is formed by combining the interference patterns obtained at different distances from the surface of the lens. A 10× magnification lens was used, and the size of the scanned area was 450 × 450 microns. The roughness coefficient was determined according to the following formula:

$$S_{q}=\sqrt{\frac{1}{N}\sum_{n\;=\;1}{\left(Z_{n}-\bar{Z}\right)}^{2}}$$

where *Z_n_* is the height of each sampling point on the scanned surface, measured from the lowest point, N is the total number of the points, and $\bar{Z}=\;\frac{1}{N}\sum_{n\;=\;1}$, Z_n_ is the average sample’s height.

The water contact angle was measured on a Kruss EasyDrop Standard instrument using DSA v1.90.0.14 software. The drop volume was 3 μL. For each sample, the contact angle was measured in three different surface areas and the results were averaged.

To study the magnetic response in surface properties of MAEs, a magnetic field was generated by permanent neodymium disk-shaped magnets. The magnet of the first type (18 × 9 × 1.5 mm^3^) is weak and produces a small magnetic field of 100 mT on the surface of a single magnet. The magnets of the second type (with a diameter of 6 mm and a height of 2 mm) produce a magnetic field of 280 mT on their surface. The magnetic field for all experiments is generated using these permanent magnets arranged in a stack. The magnetic field values are measured near the surface of the magnets using a digital Tesla meter (“Meterk”, Shenzhen, China).

**Figure 2:**
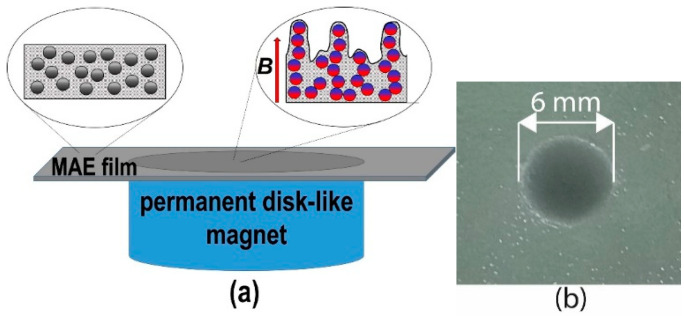
(**a**) Schematic representation of a MAE sample in the magnetic field of a permanent magnet; (**b**) photo of the MAE surface with a magnet underneath, taken in reflected light.

A MAE sample is placed on the surface of the permanent magnet (Figure 2), and the strength of the magnetic field is varied by changing the number of magnets in the stack (from one to ten). Figure 2b shows a photograph of the surface of the MAE film. A disk-shaped magnet placed directly under the film causes some changes in the surface structure, resulting in the appearance of a black circle with the size of the magnet under the film, which can be seen by the naked eye.

## 3. Results

### 3.1. Characterization of MAE Samples

Two series of synthesized MAE samples differ in the type of polymer matrix. According to the producer specification, the SP samples of the first series are based on the linear polymer network schematically shown in Figure 1a, formed upon the crosslinking of vinyl- and hydride-containing components of the silicone compound. Conversely, the SC samples contain grafted side chains within the crosslinker (Figure 1a), which comprises multiple hydride groups. It has previously been shown that the presence of side chains in polymer network subchains effectively dilutes the system and reduces the Young’s modulus of the resulting elastomer [44]. An additional reduction in Young’s modulus is achieved by the addition of plasticizer. It is important to note that even without any plasticizer both types of polymer matrices have a component that is not chemically bound to the polymer network. We estimated a fraction of such a component by performing Soxhlet extraction of the matrices. SP matrix contains 51% of sol fraction and SC matrix contains 32% of sol fraction.

To evaluate the effect of the matrix architecture and the plasticizer content, the samples for each series are prepared with the weight concentration of CIP equal to 75%, but with different concentrations of plasticizer (0, 25, 40, and 60 wt.%). To investigate the effect of the magnetic filler concentration, SC-matrix samples with 65 and 70 wt.% of CIP and 0, 25 wt.% of oil are obtained.

**Figure 3:**
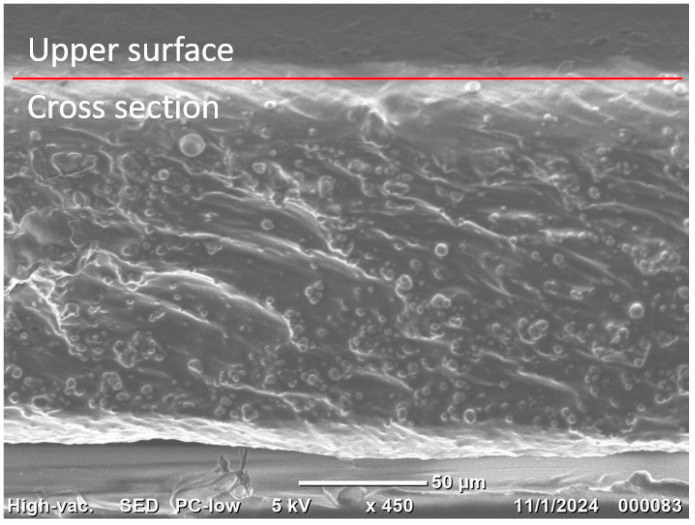
SEM image of the side of the SP MAE with 75 wt.% CIP and 40 wt.% oil.

It has been found that magnetic particles are homogeneously distributed within the polymer matrix. As an example, Figure 3 shows a cross-section of an SP MAE sample with 75 wt.% CIP and 25% oil. One can see that there is neither a noticeable depleted layer near the top nor an enriched CIP layer near the bottom of the sample.

**Figure 4:**
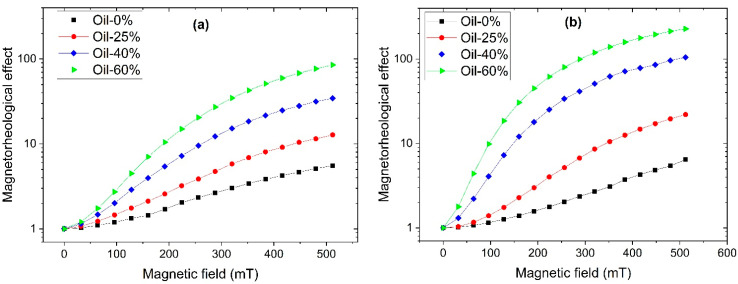
Magnetic field dependence of the magnetorheological effect for MAEs based on (**a**) SC and (**b**) SP matrices.

All the samples demonstrate a significant magnetorheological effect (MRE) which is characterized by the relative increase in the shear modulus in the external magnetic field. Figure 4 shows the magnetic field dependence of the magnetorheological effect calculated as $G_{B}^{\prime }/G_{0}^{\prime }$, where $G_{0}^{\prime }$ is the initial storage modulus of the MAE in the absence of any magnetic field and $G_{B}^{\prime }$ is the storage modulus measured in the magnetic field *B*. The values of $G_{0}^{\prime }$ are in the range of 5–18.5 kPa for SP MAEs and 3.3–15.0 kPa for SC MAEs, decreasing with the oil and increasing with CIP concentration. The magnetic field is varied from 0 to 500 mT, corresponding to the values used in the study of the surface properties of MAE films. The growth of MRE with applied magnetic field arises from enhanced magnetic interactions between particles, which tend to reorganize into chain-like aggregates aligned along the field lines [4,5,6]. The softness of the polymer matrix—reflected in its low elastic modulus—allows these magnetic interactions to overcome elastic restoring forces, enabling particle reorganization [5,6]. One can see that the higher the oil content, the higher the MRE for both MAE series. For the samples with the highest oil content of 60 wt.%, the relative increase in the shear modulus in a moderate magnetic field of 500 mT reaches 87 times for the SC series and it is higher than two orders of magnitude for the SP series. The higher MRE values observed for the SP MAEs are presumably attributed to a higher total concentration of the low-molecular-weight component, which allows for greater freedom in the restructuring of magnetic particles.

### 3.2. The Surface Roughness of MAE Samples

**Figure 5:**
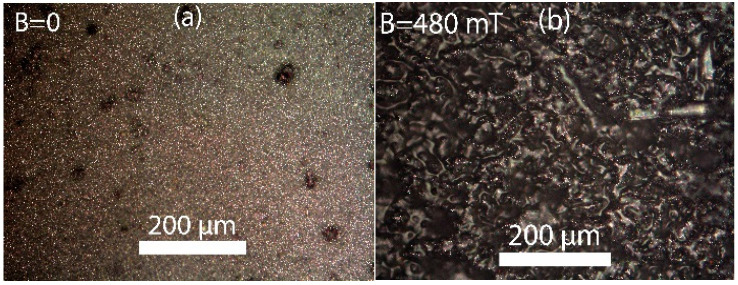
Optical images of the surface of SC MAEs with 75 wt.% CIP and 60 wt.% oil (**a**) in the absence of a magnetic field and (**b**) in the magnetic field of 480 mT.

Changes in the surface structure of MAEs under an applied magnetic field can be clearly observed using an optical microscope. As an example, Figure 5 shows optical images of an SC sample surface, which appears flat and matte in the absence of a magnetic field (Figure 5a), and becomes visibly roughened when the magnetic field is applied (Figure 5b).

**Figure 6:**
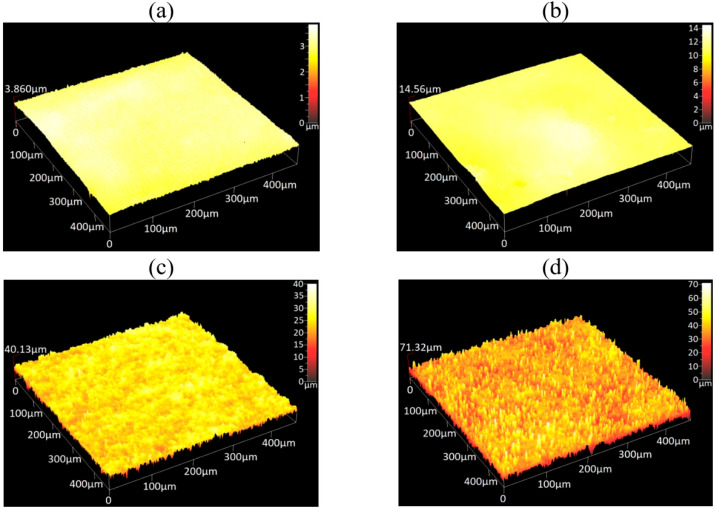
Optic profilometer images of the surface structure of SP MAEs (**a**,**c**) and SC MAEs (**b**,**d**) in the absence of a magnetic field (**a**,**b**) and in the magnetic field of 400 mT (**c**,**d**).

More details of the surface profile were obtained using an optical profilometer. Figure 6a,b presents the 3D off-field surface images of SP and SC MAEs containing 60 wt.% oil, confirming that the surface is smooth in the absence of a magnetic field. Conversely, Figure 6c,d illustrates the surface structure of the same MAE samples under a magnetic field of 400 mT, demonstrating a significant increase in surface roughness. The increase in surface roughness is caused by the formation of chain-like aggregates of magnetic particles along the magnetic field lines, which are predominantly oriented perpendicular to the sample surface, and their “growth” on the surface of compliant MAEs [23,24,25].

**Figure 7:**
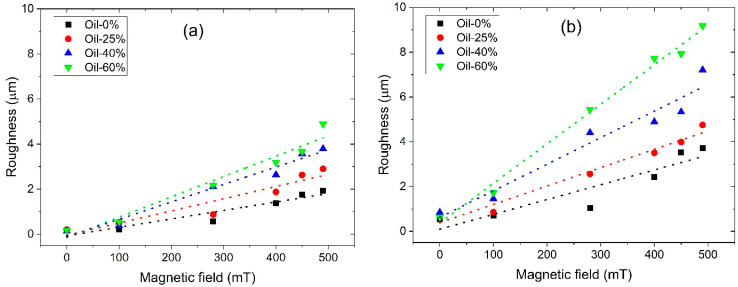
Roughness of the MAEs’ surface with 75 wt.% CIP based on SP (**a**) and SC (**b**) polymer matrix.

Figure 7 shows the magnetic field dependence of the average surface roughness for both SP (a) and SC (b) MAEs containing different concentrations of plasticizer. It should be noted that the SC samples exhibit slightly higher initial roughness at zero magnetic field compared to the SP samples, presumably due to differences in the polymer matrix structure and a lower content of low-molecular-mass plasticizer. As seen in Figure 7, the surface roughness of all MAE samples increases monotonically with increasing magnetic field, enhancing magnetic interactions and magnetic particle structuring. This general trend is in full agreement with theoretical predictions [26] and previously reported experimental data [23,25], which are explained by magnetically induced structuring of the magnetic filler into chains perpendicular to the MAE surface, resulting in the formation of a mountain-like surface relief.

The magnitude of the roughness increase correlates with the oil content: the higher the plasticizer concentration, the more pronounced the roughness enhancement under the magnetic field. This effect can be attributed to the softening of the MAEs caused by the addition of oil to the polymer matrix, which facilitates the reorganization of magnetic particles under the applied field. This result is supported by computer simulations [26] and experimental findings [24,28]. Such restructuring within the bulk material leads to an enhanced magnetorheological effect, while the growth of particle clusters on the surface gives rise to increased surface roughness.

Notably, the SC MAE samples show a more pronounced increase in surface roughness, suggesting a greater effect of the magnetic field. Surprisingly, the MRE values of the SP series are slightly higher than for the SC series MAEs (Figure 4), while the change in surface roughness is twice higher for the SC samples. Furthermore, at the maximum magnetic field, the roughness of SC MAEs with 25 wt.% oil exceeds that of SP with maximum 60 wt.% oil. The SP and SC MAEs have similar elastic moduli when the same amount of plasticizer is added. However, at the same amount of added oil, the total amount of the low-molecular-mass component is much smaller in the SC matrix. The decrease in the SC matrix’s modulus is due to the presence of side chains that are chemically connected to the polymer network. The reduction in the low-molecular-mass component, which can easily migrate inside the polymer network while maintaining material softness of side-chain MAEs, results in a more pronounced variation of surface roughness by magnetic field. One might speculate that the redistribution of oil during “magnetic mountain” growth on the surface differs between the two series, with excess oil in the SP samples smoothing out the surface relief. Indeed, in SC MAEs, the side chains are covalently attached to the polymer backbone and undergo the same microscopic deformations as the network during magnetic particle restructuring. In contrast, in SP-based composites, the mobile plasticizer can flow from the emerging surface protrusions (“mountains”) into the surrounding depressions, effectively smoothing the surface relief.

### 3.3. The Water Contact Angle of MAE Samples

Tuning the surface roughness through magnetic field application also allows for modulation of the hydrophobicity of MAEs [23,27,28]. It has been shown previously that soft PDMS-based MAEs can demonstrate superhydrophobicity in applied magnetic fields, i.e., the water contact angles (WCAs) exceeding 150° [23,24].

**Figure 8:**
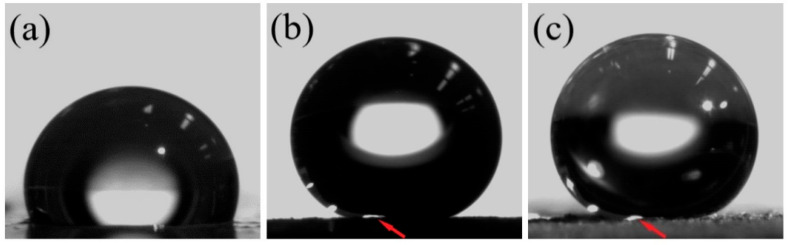
Optical images of a water droplet on the surface of MAEs with 75 wt.% carbonyl iron based on SC polymer matrix with 60 wt.% plasticizer in the absence of a magnetic field (**a**), in 490 mT magnetic field (**b**) and a water droplet image with extra lighting in the 490 mT magnetic field (**c**).

Figure 8 shows optical images of 3 µL water droplets on the surface of the SC MAE sample with 60 wt.% oil. In the absence of any magnetic field (Figure 8a), the surface of the sample is smooth and the contact line is clearly visible due to light reflection. The application of a magnetic field causes a significant increase in the contact angle (Figure 8b), and at the same time, it also causes some surface deformation due to the presence of a magnetic field gradient at the edges of the magnet. This causes some difficulties in determining the contact line, because the lowest part of the water droplet cannot be seen by the camera from the side direction. In particular, the light spot at the bottom of the water droplet is not clearly visible from the side, as shown by the arrow in Figure 8b. To observe the whole drop, the camera was tilted slightly to see the bottom part of the water droplet (Figure 8c). It is easy to see in Figure 8c that the surface of the sample in the magnetic field is very rough.

**Figure 9:**
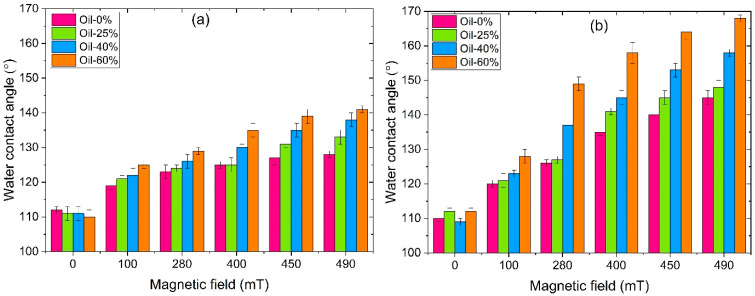
Magnetic field dependence of the water contact angle of the MAEs based on SP (**a**) and SC (**b**) polymer matrix containing 75 wt.% of CIP.

Figure 9 shows the magnetic field dependence of the water contact angle of MAEs based on SP and SC polymer matrices. The contact angle of all MAE samples increases significantly with increasing magnetic field at any plasticizer concentration. For both SP and SC MAEs, the contact angle increases with increasing oil concentration in the polymer matrix, presumably due to an enhanced mobility of magnetic particles and, thus, an increase in surface roughness in an applied magnetic field. The highest WCA values are observed for the SC MAEs with the highest oil content. For the SC sample with 60 wt.% oil, the superhydrophobicity is already reached in the magnetic field of 280 mT. The highest value of the contact angle is equal to 168° ± 1°, that is higher than those reported in Refs. [23,24]. The value of 163.0° ± 2.3° has been observed in [23] for uncured PDMS films with fluorinated carbonyl iron particles in a magnetic field of 250 mT while the value of 165° ± 2° has been observed for linear PDMS-based MAEs with 70 wt.% of CIP and 66 wt.% of plasticizer in a 600 mT field [24].

**Figure 10:**
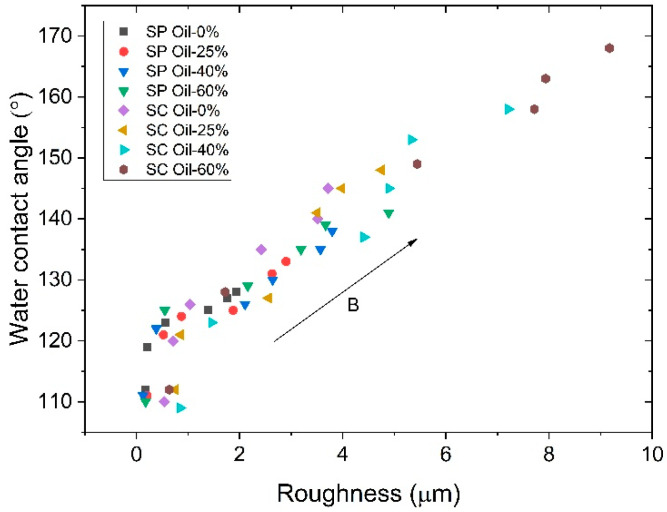
The roughness dependences of the water contact angle of the MAE samples based on SP and SC polymer matrices and containing various amounts of oil as indicated.

The roughness and contact angle data obtained for all samples in different magnetic fields are combined in Figure 10, where the contact angles are presented as a function of roughness. The data demonstrate a high degree of correlation, with the majority of points falling closely along a single master curve. After a sharp jump in wettability realized upon the application of a low magnetic field, almost a linear dependence of the WCA on the surface roughness is observed for all MAEs. So, for both series of samples, the surface roughness is the primary factor affecting the wettability of the MAE surface.

**Figure 11:**
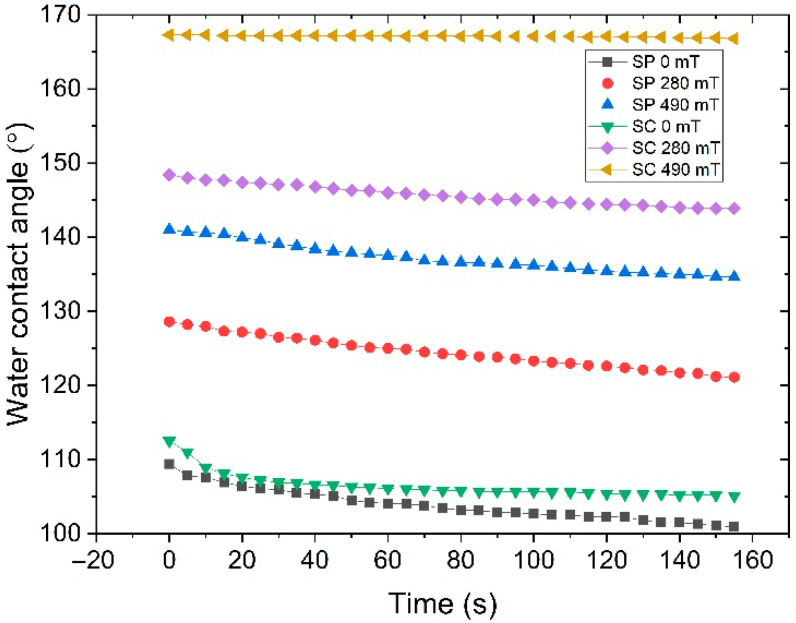
The time dependence of the water contact angle for SP and SC MAEs containing 75 wt.% CIP and 60 wt.% oil, measured in different magnetic fields.

Figure 11 shows the time dependence of the water contact angle of SP and SC MAE samples containing 60% oil and 75% filler particles, as measured in various magnetic fields. The contact angle of water droplets is strongly influenced by water evaporation [22,36]. In the absence of a magnetic field, the WCA decreases by 10% on the SP MAE surface within 2.5 min. The WCA on the SC MAE surface is more stable; it decreases within 30 s but remains almost constant over time. For both series, the WCA achieved at higher magnetic fields exhibited greater stability over time compared to the WCA in the absence of a magnetic field. However, comparing the time dependence of the WCA for the SP and SC MAE samples reveals that the WCA is more stable for the SC MAEs. In a 490 mT field, the WCA on the SC MAE surface remains at 168°, while the WCA on the SP MAE surface decreases from 142° to 135°.

The reported results show that not only the elastic modulus but also the network architecture is a powerful tool to control the surface properties of MAEs. Introducing side chains into the polymer matrix reduces the low-molecular-weight fraction while maintaining composite softness, thereby enabling effective reorganization of magnetic particles under an applied magnetic field. On the other hand, the reduced low-molecular-weight fraction in side-chain matrices enhances surface property modulation by suppressing local redistribution of matrix components during polymer deformation in the course of magnetic filler restructuring in a magnetic field because the side chains are chemically grafted to the network subchains and move together with them. This results in a higher surface roughness, and higher values of WCA and their stability.

**Figure 12:**
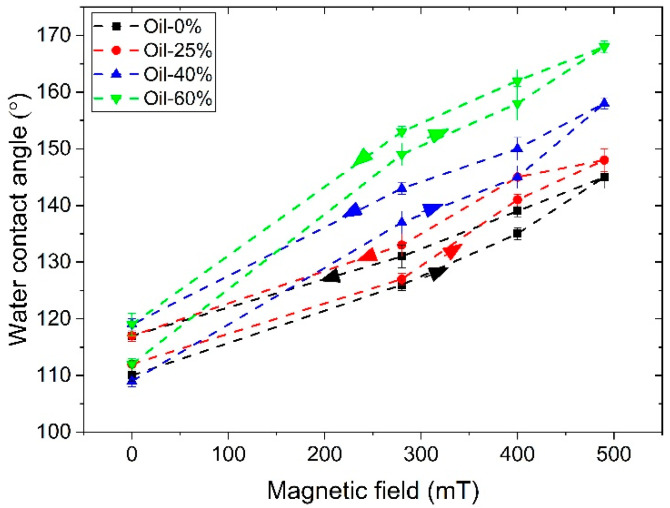
Hysteresis of the water contact angle of SC MAE samples with 75 wt.% of CIP.

It is well established that MAEs are characterized by long relaxation times [48,49,50,51]. In particular, for the linear PDMS samples, it has been found that the rearrangement processes of the filler is characterized by three characteristic time constants differing approximately by one order of magnitude: τ_1_~10^1^ s, τ_2_~10^2^ s, and τ_3_~10^3^ s. In this study, the contact angle of MAE samples based on the SC polymer matrix was first measured while increasing the magnetic field up to its maximum value, followed by a measurement during the subsequent decrease in the field. Notably, higher contact angle values were observed during the decreasing field phase, or “reverse” branch of the curve (Figure 12). This behavior resembles magnetic hysteresis, although it should be noted that carbonyl iron particles are magnetically soft and typically exhibit negligible intrinsic magnetic hysteresis. The observed hysteresis phenomenon can be attributed to residual surface roughness that remains after the application and removal of the magnetic field due to long relaxation times of this material. As a result, the contact angles measured during the decreasing field phase were consistently higher than those recorded during the increasing phase.

Additionally, we studied the effect of CIP concentration on the WCA of the SC MAE films. Table 2 summarizes the average WCA values measured for SC MAEs containing 65, 70, and 75 wt.% CIP, along with 0 wt.% and 25 wt.% plasticizer. In all cases, the WCA increases under an applied magnetic field. Even the sample with the lowest filler content exhibits a clear magnetic response. As expected, increasing the CIP concentration enhances the magnitude of WCA change in the presence of a magnetic field.

Previously, results for MAE samples with a thickness of 0.2 mm were discussed. The water contact angles of all MAE samples with a thickness of 0.4 mm were also measured. It was found that the contact angles of the 0.2 mm thick samples were higher than those of the 0.4 mm thick samples. This difference can be attributed to a weaker magnetic field at the surface of the thicker samples, as the distance between the permanent magnet and the MAE surface increases with sample thickness.

**Figure 13:**
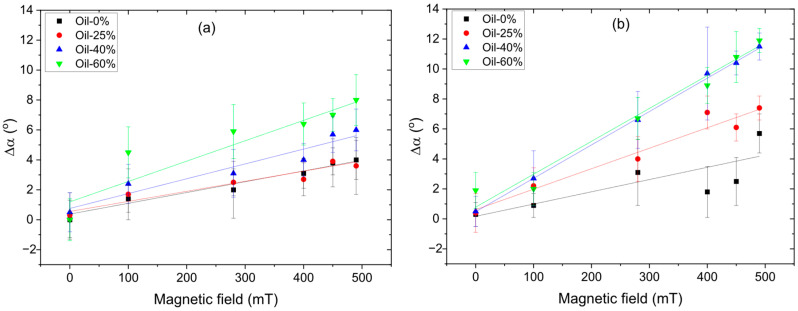
The difference between water contact angles of the SP (**a**) and SC (**b**) polymer matrix-based MAE samples with 0.2 mm and 0.4 mm thickness.

The difference in contact angles between individual MAE samples of 0.2 mm and 0.4 mm thickness was calculated for the same oil concentration. Figure 13 presents this difference in water contact angles for MAEs of different thicknesses h: Δα = α(h = 0.2 mm)-α(h = 0.4 mm) where α is the water contact angle. It can be seen that Δα increases with increasing magnetic field for all samples. Additionally, MAE samples with higher oil content exhibit larger Δα values. The close agreement between the SC MAE curves for 40% and 60% of oil may be attributed to measurement uncertainty or could indicate a saturation effect in the system at higher oil contents. Furthermore, SC MAE samples show higher Δα values compared to SP MAE samples. However, despite the 10–12° decrease in WCA with the film width, WCA values still remain higher than 150° in the maximum magnetic field.

## 4. Conclusions

In this paper, we present the results of a comparative experimental study on the surface structure and water contact angles (WCA) of magnetoactive elastomers (MAEs) based on two types of polymer matrices. The first type of elastomer was prepared from a commercially available silicone compound, which forms a linear polymer network upon chemical crosslinking (referred to as SP matrix). The second type was synthesized from a mixture of divinyl- and monovinyl-terminated silicones together with a multifunctional hydride-containing silicone crosslinker, resulting in a polymer network with multiple side chains (referred to as SC matrix). MAEs containing 75 wt.% carbonyl iron microparticles and varying amounts of low-molecular-weight silicone oil were fabricated using both matrix types. Additionally, the effect of magnetic filler content was investigated for MAEs with the SC matrix.

It has been demonstrated that

- MAEs exhibit a giant magnetorheological effect, with the elastic modulus increasing by more than two orders of magnitude. SP-based MAEs show a stronger magnetorheological response.
- MAEs display significant magnetic field-induced changes in surface properties. Surface roughness reaches nearly 10 μm, and water contact angles (WCA) reach up to 168°. SC-based MAEs show more pronounced changes in surface properties.
- WCA data for all MAE samples collapse onto a single master curve when plotted as a function of surface roughness, revealing a clear linear relationship.
- WCA exhibits hysteresis during increasing and decreasing magnetic field cycles, indicating memory effects in surface restructuring.
- WCA gradually decreases over time, with this decline being more pronounced for the SC-based MAE series.
- Increasing the thickness of MAE films from 0.2 mm to 0.4 mm reduces the WCA, likely due to a lower effective magnetic field at the surface, farther from the field source. Nevertheless, WCA for SC-based MAEs still reaches values as high as 150°, indicating strong superhydrophobic behavior.

The most important conclusion that can be drawn from the obtained results is that network architecture is a powerful tool for controlling the surface properties of magnetoactive elastomers. The incorporation of side chains into the network structure not only reduces the amount of leachable low-molecular-weight components but also enhances the extent of magnetic field-induced surface modulation. These findings are expected to be valuable for the development of smart surfaces with tunable wettability, particularly for applications in soft robotics and other fields requiring responsive materials.

## Figures and Tables

**Table 1:** Characteristics of components used in the synthesis of polymer matrices and their content in the matrix.

Component	Chemical Formula	M_w_	M_w_/M_n_	m, g
	**Matrix 1**			
α,ω-divinyl-polydimethyl-siloxane	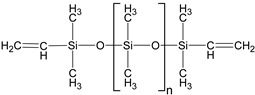	37,000	1.67	5
Polymethyl-hydrosiloxane	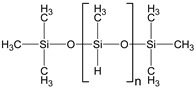	3600	2.21	0.0446
α,ω-dihydride-polydimethyl-siloxane	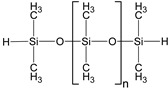	1600	1.38	0.1621
ω-vinyl-polydimethyl-siloxane	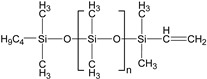	3100	1.11	1.8153
	**Matrix 2**			
Vinyl-containing silicone	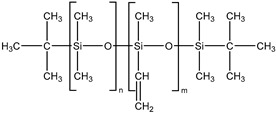	100,000	1.43	3
Vinyl- and hydride-containing silicone	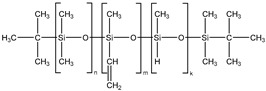	100,000	1.43	3

**Table 2:** The water contact angles for SC MAEs with different concentrations of CIP.

Magnetic Field, mT	Contact Angle α, °					
	65 wt.% CIP		70 wt.% CIP		75 wt.% CIP	
	0% Oil	25% Oil	0% Oil	25% Oil	0% Oil	25% Oil
0	111 ± 1	109 ± 2	111 ± 2	110 ± 1	110 ± 1	112 ± 1
100	120 ± 1	121 ± 2	120 ± 2	121 ± 2	120 ± 1	121 ± 2
280	126 ± 1	127 ± 1	128 ± 1	127 ± 2	126 ± 1	127 ± 1
400	134 ± 2	135 ± 1	136 ± 2	139 ± 1	135 ± 1	141 ± 1
450	137 ± 1	138 ± 1	139 ± 1	139 ± 2	140 ± 2	145 ± 2
490	138 ± 1	140 ± 2	141 ± 2	143 ± 2	145 ± 2	148 ± 2

## Data Availability

Data are contained within the article.
